# Achieving a timely diagnosis for teenagers and young adults with cancer: the ACE “too young to get cancer?” study

**DOI:** 10.1186/s12885-019-5776-0

**Published:** 2019-06-24

**Authors:** Rachel M. Dommett, Hannah Pring, Jamie Cargill, Paul Beynon, Alison Cameron, Rachel Cox, Aoife Nechowska, Alison Wint, Michael C. G. Stevens

**Affiliations:** 1 South West TYA Cancer Service, Bristol Haematology Oncology Centre, University Hospitals Bristol NHS Foundation Trust, Horfield Road, Bristol, BS2 8ED UK; 2 Department of Paediatric Haematology Oncology & BMT, Bristol Royal Hospital for Children, University Hospitals Bristol NHS Foundation Trust, Bristol, UK; 3 Macmillan GP and NHS Bristol, North Somerset & South Gloucestershire CCG, Bristol, UK; 4 Translational Health Sciences, Bristol Medical School, University of Bristol, Bristol, UK

**Keywords:** TYA, Time to diagnosis, Routes to diagnosis, Primary care, Secondary care

## Abstract

**Background:**

Time to diagnosis (TTD) concerns teenagers and young adults (TYA) with cancer and may affect outcome.

**Methods:**

Healthcare records from 105 TYA in a regional cancer service were assessed to document events from 1st symptom to treatment start. Detailed pathway construction was possible for 104 patients and allowed a multidisciplinary panel review of each pathway with assessment of good practice and lessons for the future.

**Results:**

1st presentation was to primary care in 86, and 93% consulted in primary care before diagnosis. Routes to Diagnosis were 45% via urgent 2 Week Wait pathways and 38% as emergency referrals. Total Interval (time from 1st presentation to treatment start) was median 63 (range 1–559) days, varying within/between diagnoses. Patient interval (time from 1st symptom to 1st presentation) was longest for lymphoma, carcinoma and bone tumour (medians: 9, 12, 20 days). Overall, time in primary care was short (median 3, range 0–537 days) compared to secondary care (median 29, range 0–195 days) and longest for lymphoma, carcinoma, brain/CNS (medians: 10, 15, 16 days). Specialist Care interval (time from 1st specialist visit to treatment start) was longest for bone, brain/CNS, lymphoma, carcinoma (medians: 30, 33, 36, 48 days). 40% pathways were rated as showing good/best practice but 16% were less than satisfactory. Continued safety-netting/support was identified from primary care but analysis suggested opportunities for improvement in transition through secondary care.

**Conclusions:**

Previous reports of prolonged TTD have focused on delay in referral from primary care but this study suggests that this might be reduced by optimising management in secondary care.

## Background

Cancer is the commonest cause of disease-related death in Teenagers and Young Adults (TYA - age 15 to 24 years) [1]. Timely diagnosis is a major concern for young people and their families [2–6] and despite limited data to link prolonged time to diagnosis (TTD) with adverse survival [7–9], actual or perceived delay may significantly impact patient experience, quality of survival, psychological health and future trust in healthcare.

Factors influencing the length of the diagnostic pathway are complex. Cancer has been considered to be a rare event in children and young people and there may still be an under-recognition of the possibility of its diagnosis amongst health professionals (“too young to get cancer?”). However, the incidence of cancer in young people age 15–24 years in the United Kingdom rose by 33% between 1992 and 95 and 2013–15 to an age standardised incidence rate of 298/million; and the cumulative risk of a diagnosis of cancer in the TYA age span now approaches 1 in 200 individuals [10]. Issues may also arise in relation to the non-specific nature of symptoms associated with the most frequently encountered diagnoses encountered by TYA and the difficulties some young people experience in navigating the health care system [2, 11–14].

Work to map the diagnostic journey (Routes to Diagnosis – RTD) for adults with cancer has highlighted variability in Time to Diagnosis (TTD) between diagnostic groups [15] and suggests that longer TTD may reduce survival [16]. Particular emphasis has been placed on strategies to improve the recognition of cancer in primary care [17–19] and attention paid to the positive predictive value of ‘alert’ symptoms [20]. Conclusions of such studies may not be easily transferrable to young people: although analysis of pre-diagnostic consultations shows positive associations between subsequent cancer diagnosis and both consultation frequency and alert symptoms, the resulting positive predictive values are too low to be of clinical value [21–24].

Specific studies exploring RTD and TTD within the TYA age range are limited. Recently published data from the BRIGHTLIGHT cohort, a study of TYA patients recruited across England, correlated patient-derived recall of symptom onset and healthcare consultation with date of diagnosis from cancer registry records [25] and highlighted that sociodemographic factors and tumour type significantly influenced pre-referral primary care consultation rates and TTD. The relationship between TTD and cancer type in TYA has also been reported by others [13, 26]. Other studies suggest low utilisation of the 2 Week Wait (TWW) referral pathway in TYA, and an excess of emergency referral [27] but, in contrast to adult data [28], there is currently no evidence that emergency referral for TYA adversely affects survival. Processes in secondary care may also contribute to increased TTD [7] but have not been formally studied in TYA.

## Methods

### Aim, design and setting

The “Too Young To Get Cancer?” study was a service evaluation project initiated within the ACE (Accelerate, Coordinate, Evaluate) programme established in England in 2014 with support from NHS England, Cancer Research UK and Macmillan Cancer Support [29]. The objective was to better understand referral pathways in a cohort of newly diagnosed patients referred to a regional care network for TYA cancer in the South West of England. Existing referral policies were in line with those mandated by the specification for TYA cancer services set within NHS England. Time to diagnosis had been identified as a priority concern by patients involved in previous service development within the network [30].

### Patient recruitment, permissions and access to healthcare records

All newly diagnosed patients referred within the regional TYA cancer care network between October 2014 and April 2016 were eligible but were approached only after current status had been verified by a TYA Clinical Nurse Specialist (CNS). Written permission to access healthcare records was obtained from all verified patients (or from next of kin if deceased).

The Caldicott Guardians^1^ (individuals responsible for protecting the confidentiality of personal data) at each NHS hospital Trust^2^ participating in the TYA network agreed to the extraction of data from hospital records. Access to primary care records was arranged with the consent of each patient’s general practitioner (GP). The inclusion of records from deceased patients was attempted to avoid bias. Next of kin were approached by the healthcare professional identified to have the closest relationship and written permission to access health care records was obtained for all deceased patients included in the study.

### Data collection

All records in primary and secondary care were reviewed by one investigator (HP) using a pro forma constructed by clinical members of the study team based on experience of patient diagnostic pathways acquired from their clinical experience.

The aim was to collect both qualitative and quantitative data about each patient’s pathway (including free text to provide context and detail) so that the entire route to diagnosis could be articulated as a coherent story, yet also summarised as a structured chain of events. These events included dates of all the following: consultations (including any scheduled but which did not take place); referrals; investigations; non-cancer treatments; diagnosis; multidisciplinary team (MDT) discussions; and first cancer treatment. Free text detail permitted inclusion of information such as: who initiated each event; what type of event it was (e.g. emergency or routine referral); where each event took place (e.g. primary care, hospital emergency department, hospital outpatient clinic); symptomatology recorded; and the outcomes or action reported (e.g. safety netting, planned review, investigation, referral).

Date of diagnosis was defined as the date the specimen resulting in the first histological or cytological confirmation of malignancy was taken.

The data abstracted from the clinical records were sorted chronologically into a bespoke database created specifically to manage these data and which included the facility to link comments and free text observations to each recorded event.

### Individual pathway maps and panel reviews

The data collected for each patient was used to construct an individual pathway map which summarised all events against a timeline (in days) and identified transitions between primary and secondary care. Key events were as described in the Aarhus Statement [31] and defined intervals along the diagnostic pathway were as described by Oleson et al. [32].

Two exemplar pathway maps are shown as Tables 1 and 2. In each case, the shaded boxes represent all events identified in relation to specific dates in the patient’s route to diagnosis. Events significant to the understanding of the pathway are identified as a short summary statement but, for reasons of space, full details are not shown. Note that the days of each pathway shown are not consecutive (also for reasons of space) but that full details of each event and every date (with the corresponding day of the week) were available in the versions presented for panel discussions.

**Table 1:**
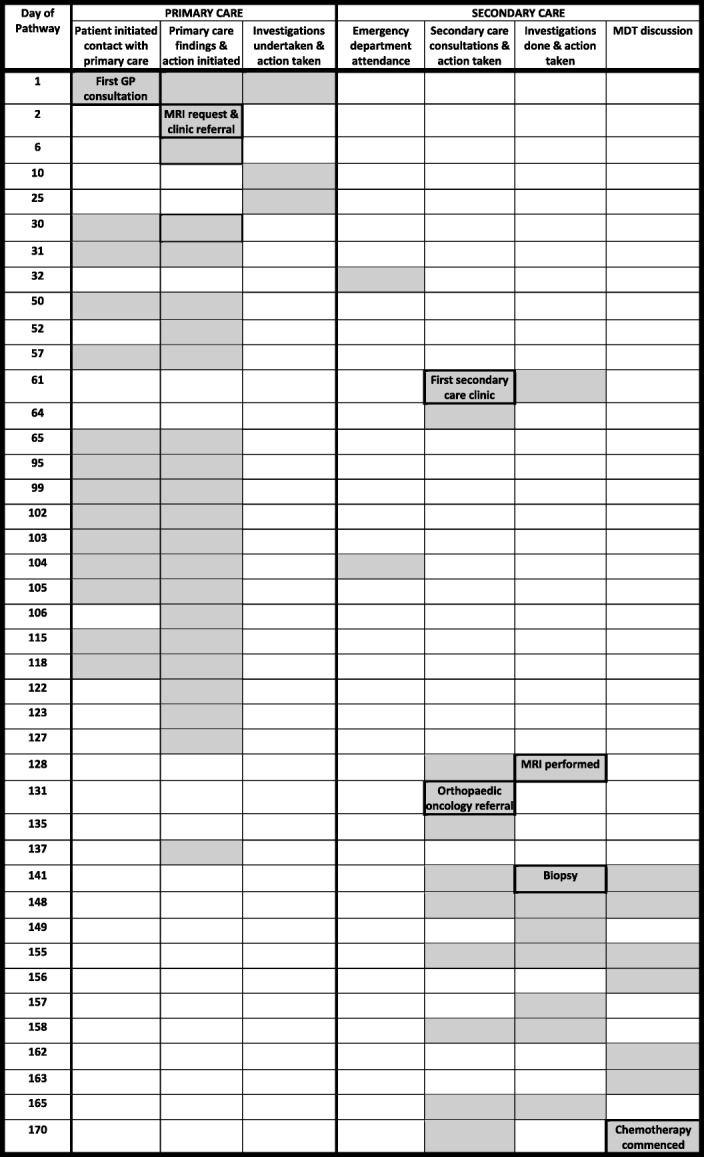
Example pathway 1 (Male, age 19 years at diagnosis of Ewing’s sarcoma) and explanatory narrative

History: Presented to GP with a 6 month history of lumps in the groin and complaining of pain in the lower back for up to 2 years. He had a previous history of recreational drug use. He was found to have inguinal lymphadenopathy and was tender over the lumbosacral spine

Pathway: Blood tests were obtained and an MRI scan was requested and referral made to a local musculoskeletal clinic on Day 2. In the meantime he attended his GP surgery on multiple occasions for pain management. A plain x ray of the hip was obtained after attending the musculoskeletal clinic (day 61) and a non-urgent referral made to the orthopaedic clinic. This did not take place until day 128 when urgent arrangements were made for MRI (which confirmed a large pelvic tumour) and CT chest (which showed metastases). Transfer was requested to a surgical orthopaedic oncology centre where a biopsy was obtained on day 141. Staging investigations and multiple MDT discussions took place, initially because of concern that re biopsy might be required. These involved both institutions and a national MDT. Chemotherapy was commenced on day 170. Throughout this time, he continued to attend his GP surgery for pain management

Intervals:

Total interval (Primary care interval (1st seen to 1st referral) = 6 days

Secondary care interval (1st referral to start of treatment) = 164 days

Diagnostic interval (1st seen to diagnosis (date of biopsy)) = 141 days

Treatment interval (Diagnosis (date of biopsy) to start of treatment) = 29 days

Key points arising in the panel discussion:

1. Primary care: despite early referral to secondary care, an appointment for the musculoskeletal clinic was not followed up despite continuing attendance for pain control; nor was the early request made for an MRI expedited despite the severity of symptoms. The patient’s previous history of drug abuse may have affected judgement about his analgesic requirement

2. Secondary care: retrospectively, it was apparent that there had been a failure to recognise an abnormality on the plain x ray obtained at the musculoskeletal clinic and further assessment at an orthopaedic clinic was not prioritised, resulting in a 67 day delay before the patient was seen and appropriate radiology obtained. Despite rapid onward referral to a specialist orthopaedic oncology centre for biopsy, a further 29 days passed from the date of biopsy to the start of chemotherapy. It was felt that staging investigations and MDT discussions should all have been achieved more quickly, particularly as the patient had metastatic disease when the diagnosis was established

“Clinical bottom line” – panel decision: Less than satisfactory

**Table 2:**
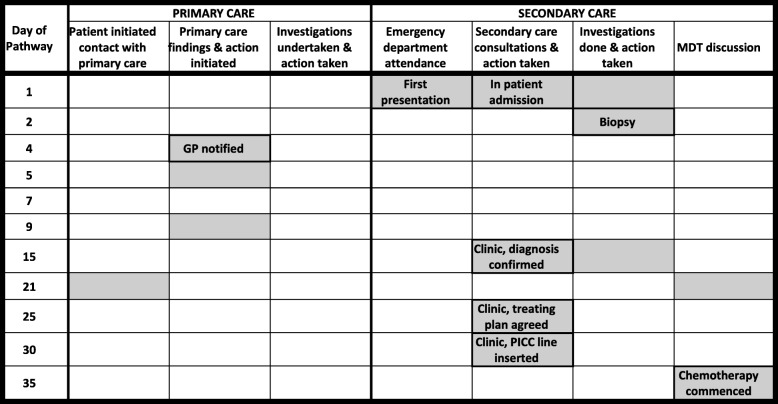
Example pathway 2 (Male, age 23 years at diagnosis of Hodgkin’s lymphoma) and explanatory narrative

History: Presented to a local emergency department with a 6 week history of feeling unwell, with cough, evolving cervical lymphadenopathy and recent onset of night sweats. He had had no prior contact with primary care and was living away from home at the time

Pathway: Patient was admitted from the emergency department, radiology and blood tests taken and a biopsy was performed the following day which confirmed Hodgkin’s Lymphoma. The patient elected to return home for treatment. His GP was informed on Day 4 and a referral made to his local hospital where he was seen as an outpatient for reassessment and completion of staging investigations on Day 15. The diagnosis and treatment plan were discussed at the local MDT on Day 21 and chemotherapy commenced on Day 35 after further discussion with the patient and insertion of a PICC line

Intervals:

Primary care interval (1st seen to 1st referral) = Not applicable

Secondary care interval (1st referral to start of treatment) = 34 days

Diagnostic interval (1st seen to diagnosis (date of biopsy)) = 2 days

Treatment interval (Diagnosis (date of biopsy) to start of treatment) = 33 days

Key points arising in the panel discussion:

1. This patient presented as an emergency and was diagnosed without delay. The time taken to commence treatment was longer than might otherwise have been necessary only because he was living away from home and elected to be referred back to his local hospital for completion of staging, treatment planning and treatment

“Clinical bottom line” decision: Best practice

All pathway maps were reviewed by a panel of senior clinicians including consultants in adult, TYA and paediatric haematology/oncology, a senior TYA nurse and a general practitioner. The aim of the panel review was to confirm key events in each pathway and to record examples of good practice; missed opportunities for potential earlier diagnosis; and whether, and at which point, an intervention to effect earlier diagnosis might have been possible. The panel also sought to identify potential interventions that could have improved transition through the pathway and/or improve patient experience.

The first date of presentation to primary care was identified by agreement within the panel as the time point at which the patient first presented with symptoms which, in retrospect, were likely to be related to their final cancer diagnosis.

The panel’s assessment was guided by a set of pre determined criteria relating to each component of the diagnostic pathway (Table 3). These criteria were established and agreed by the panel prior to starting work on pathway review. They were based on a consensus view of issues that might be relevant to decisions taken along the route to cancer diagnosis and were informed by the panel members’ own clinical practice and experience.

**Table 3:** Criteria taken into consideration by panel in assessing each event in the pathway

	Initial diagnostic assessment	Diagnostic test performance and interpretation	Diagnostic follow up and consultation	
			Primary care	Secondary care
Patient	Language. Geography. Comorbidity. Psycho-social factors.	Non adherence	Passive/Active FU of results. Unsafe-safety netting. Inconsistent symptoms or resolution of symptoms.	Passive/Active FU of results. Unsafe-safety netting. Inconsistent symptoms or resolution of symptom
Healthcare Professional	Inadequate history and/or examination. Cognitive factors, unfamiliarity with cancer presentation. Comorbidities. Referral norms. Continuity of care.	Misinterpretation of results, false negative. Lack/delay of FU of results. Deficient investigation strategy/wrong test. Communication.	Over reliance on patient to re-present. Timely FU. Communication. Coordination failures. Lack of appreciation or FU of abnormal test result. Continuity of care.	Over reliance on patient to re-present. Timely FU. Communication. Coordination failures. Lack of appreciation or FU of abnormal test result. Continuity of care.
System	Rigid consultation norms. Access/system capacity constraints. Access to diagnostic tests. Administrative failure in booking	Lack of system to deal with failure to attend. Diagnostic testing process complexity. Lack of ownership of results.	Accountability for the patient as they progress through the diagnostic pathway.	Accountability for the patient as they progress through the diagnostic pathway.
Disease	Atypical symptoms and/or presentation.	False negatives.		

**Fig. 1:**
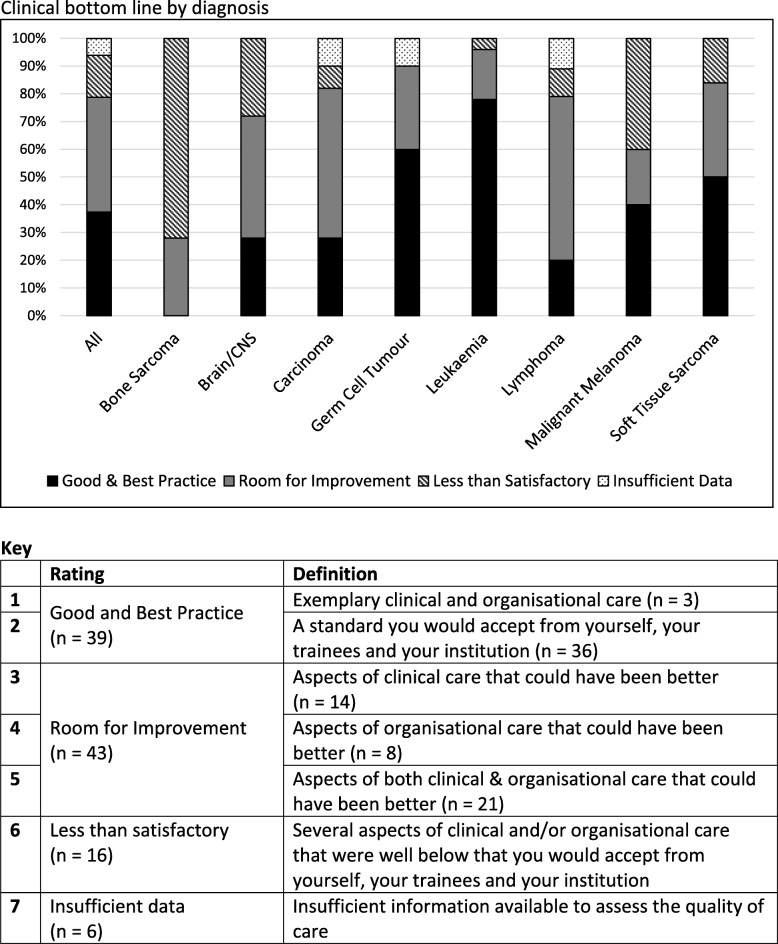
Clinical bottom line by diagnosis

A final outcome (“clinical bottom line”) was discussed by the panel for each pathway and documented as its consensus view, using an overall rating statement similar to that used by the National Confidential Enquiry into Patient Outcome and Death (NCEPOD) [33] and shown in the legend to Fig. 1.

### Outcomes and feedback

The study protocol did not include a plan to collect or analyse feedback received from the dissemination of the study’s findings although outcomes and lessons learned from the study were shared in a number of settings. This included a dissemination event to which all those who had participated (professionals, patients and next of kin) were invited; to staff at the hospitals in which patient records were accessed; to primary care staff at a regional information cascade event; to the TYA community at national and international meetings; and to other ACE programme participants.

**Fig. 2:**
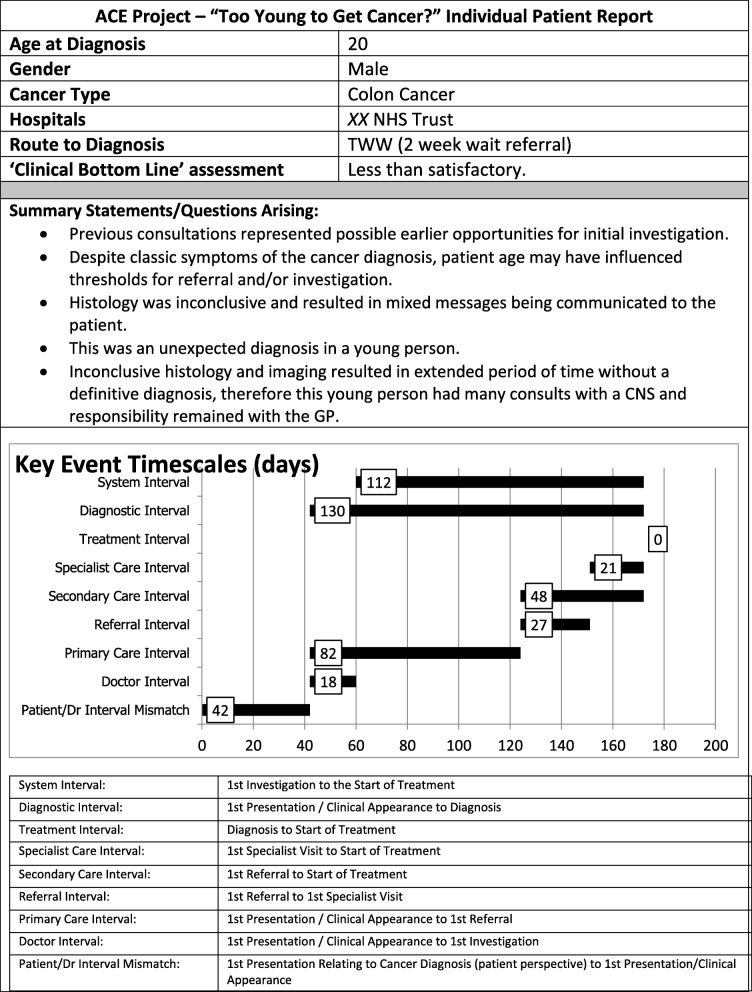
Front page from a patient summary report prepared for clinicians involved in care

Reports of individual patient pathways were constructed for sharing with the clinicians involved. An anonymised extract of the front page of one such report is shown as Fig. 2: note that each report was accompanied by a narrative explanation of factors considered relevant to the patient’s transition through the pathway and to the ‘Clinical Bottom Line’ conclusion reached by the panel.

### Statistical analysis

As conclusions were based on data obtained from an audit of individual patient records across a range of different diagnoses without a comparator group or intervention, formal statistical analysis was utilised only to compare responders who allowed their records to be accessed with those who did not (Chi squared test). Simple descriptors were used to aggregate data into broad groups by diagnosis, age and gender. Pathway intervals were expressed as median and range.

### Ethical approval

In line with guidance from the NHS Health Research Authority which confirms that formal ethical review is not required for audit and service evaluation [34], formal research ethical approval was not requested. All TYA eligible for the study were nevertheless approached with written information about the purpose of the study and written consent was required to access health care records.

## Results

### Patients

186 patients were identified as potentially eligible for the study of whom 166 (89%) were approached to participate; 20 patients were not approached based on the judgement made by the relevant TYA CNS although reasons were not recorded. Permission was received from 105/166(63%) but data from one patient was considered insufficient to evaluate the pathway. The characteristics of the 104 patients included in the study were: 54(52%) male; 34(33%) aged 15–18 years; and 97(93%) self-identified as white British. Diagnoses were: Lymphoma 29(28%); Carcinoma 21(20%); Leukaemia 18(17%); Germ cell tumours 10(10%); Brain/CNS tumours 7(7%); Bone tumour 7(7%); Soft tissue sarcoma 6(6%); Melanoma 5(5%); and ‘other’ 1 (Wilms’ tumour). Comparison with the 61 non-responders to the invitation showed that response rates varied by diagnosis (highest (> 70%) amongst those with lymphoma, bone tumour and carcinoma and lowest (< 50%) in those with germ cell tumour and melanoma) but only a deficit in the number of patients with germ cell tumours in the responder group reached statistical significance (*p* < 0.02). Three patients were included in the study after their death with the permission of their next of kin.

### Data collection, pathway preparation and panel review

Data were collected from 66 GP practices and from 9 hospital Trusts over a 10 month period ending in September 2016. The complexity of pathways varied widely; for example, some only included 10 events, whereas in one patient 69 events were identified. All pathways were discussed by the panel using the criteria shown in Table 3. The panel convened nine times; on average, 25 min was required for the evaluation and discussion of each pathway, although in complex cases this took up to 1 h.

### Patient pathway analysis

The first presentation relating to the cancer diagnosis was to primary care in 86%; 7% to hospital Accident & Emergency (A&E) departments; 4% to other healthcare professionals; and 3% unknown/unclear. Overall, cancer was suspected at first presentation in 34% patients and varied by diagnostic group - highest in germ cell tumours (67%) of whom 9/10 were men presenting with testicular mass. Cancer was not suspected at first presentation in any patient (*n* = 7) with bone sarcoma. (Table 4).

**Table 4:** Place and suspicion of cancer at first presentation, by diagnosis

Diagnosis	Pathway data (n)	Primary Care (n)	A&E (n)	Other (n)	Unknown (n)	Cancer suspected (%)
Lymphoma	29	25	3	0	1	44
Carcinoma	21	16	0	3	2	21
Leukaemia	18	16	2	0	0	33
Germ Cell	10	9	0	0	1	67
Brain / CNS	7	5	1	1	0	14
Bone Tumour	7	6	1	0	0	0
Melanoma	6	6	0	0	0	60
Soft Tissue	5	5	0	0	0	33
Other	1	1	0	0	0	0
Total	104	89 (85.6%)	7 (6.7%)	4 (3.8%)	4 (3.8%)	Overall 34%

#### Primary care

**Fig. 3:**
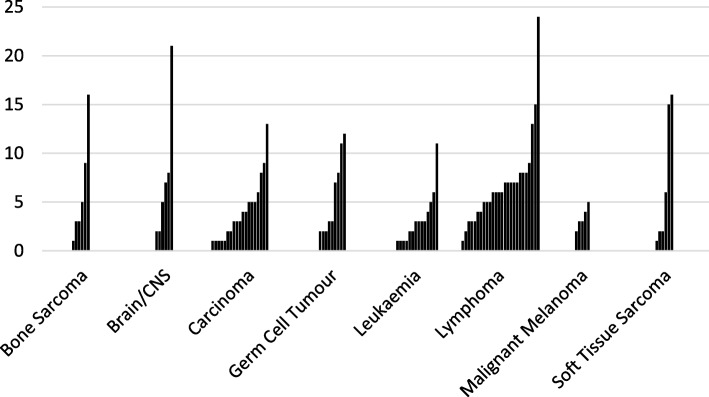
Number of primary care contacts from 1st presentation to start of treatment by type of cancer diagnosis i.e. throughout the Total Interval (each bar represents one patient)

Whilst 86% had consulted primary care prior to referral to secondary care, 93% consulted at some point prior to diagnosis, many on multiple occasions. Some patients continued to consult in primary care after diagnosis and up to the point of treatment. The frequency and variability of primary care consultation is shown in Fig. 3; for example, 15/25(60%) patients with lymphoma who had consulted primary care had done so ≥5 times before start of treatment. Patients also accessed out of hours services, used telephone consultations and presented to A&E departments, sometimes before consulting their own GP.

#### Routes to diagnosis

Using established RTD definitions [14], 45% of patients were referred from primary care via urgent 2 Week Wait (TWW) pathways; 38% emergency referrals; 11% routine referrals; 6% other outpatient routes; and 1 patient via screening.

**Fig. 4:**
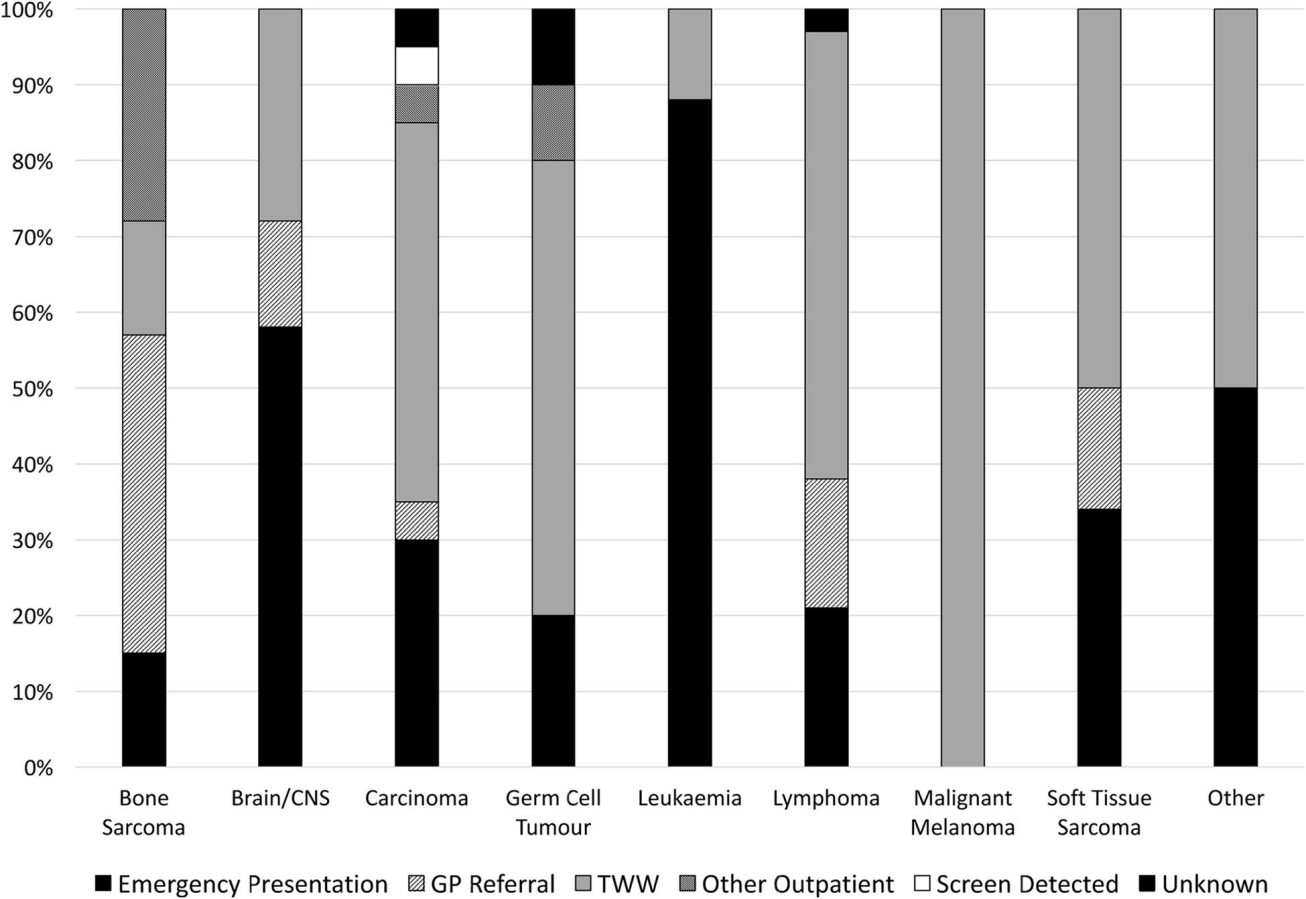
Routes to Diagnosis by cancer type

RTD varied by diagnostic group (Fig. 4). All malignant melanoma patients and over half of lymphoma patients presented via TWW compared with only 1/7 bone sarcoma and 0/7 of the brain/CNS tumour patients.

Of the 40 (38%) patients who presented as emergencies, 16 had leukaemia (89% of all leukaemia patients). In considering diagnoses other than leukaemia, 22/86 (26%) presented as emergencies. Those with brain/CNS tumours (4/7, 57%), carcinoma (6/21, 29%) and lymphoma (6/29, 21%) were the largest contributors to this group. Overall, 37% of all patients referred via an emergency route were considered by the panel to have had opportunities for an earlier referral by another route.

#### Total interval

**Fig. 5:**
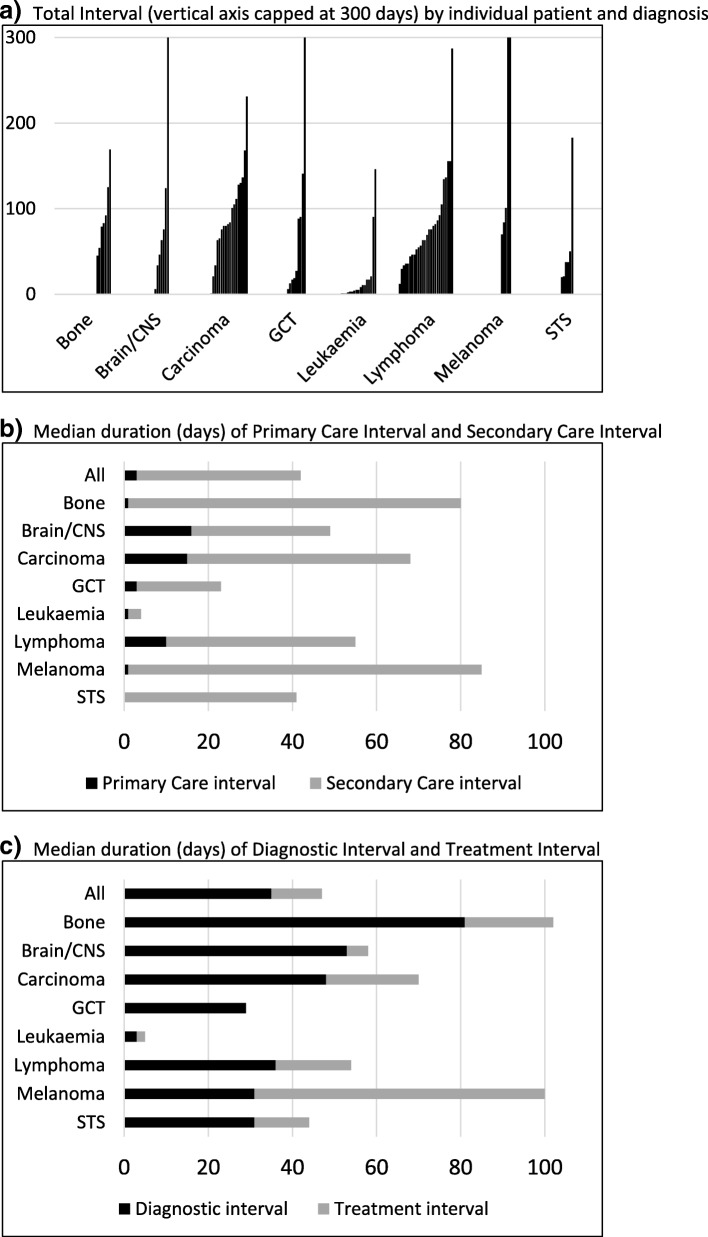
**a** Total Interval (vertical axis capped at 300 days) by individual patient and diagnosis. **b** Median duration of Primary Care Interval and Secondary Care Interval (days). **c** Median duration of Diagnostic Interval and Treatment Interval (days)

Table 5 shows median duration of each interval by diagnosis. The longest total interval (time from first presentation to start of treatment) was in patients with melanoma (median 99 days (range 70–392)) and bone sarcoma (86, (45–169)), and the shortest intervals in patients with leukaemia (7 (1–146)) and germ cell tumours (29 (6–559)) but with variability between and within each diagnostic group (Fig. 5a).

**Table 5:** Median (range) days for each interval by diagnosis

Diagnosis (No.)	Total Interval	Patient Interval	Doctor Interval	Primary Care Interval	Referral Interval	Secondary Care Interval	Specialist Care Interval	Diagnostic Interval	System Interval	Treatment Interval
All diagnoses (104)	63 (1–559)	0 (0–417)	3 (0–537)	3 (0–525)	7 (0–83)	39 (1–231)	29 (0–195)	35 (0–559)	49 (0–287)	12 (0–111)
Lymphoma (29)	63 (12–287)	9 (0–334)	1 (0–73)	10 (0–76)	7 (0–83)	45 (3–147)	36 (3–132)	36 (1–131)	61 (3–287)	18 (0–93)
Carcinoma (21)	81 (1–231)	12 (0–267)	14 (0–71)	15 (0–27)	7 (0–36)	53 (1–231)	48 (1–195)	48 (1–231)	72 (0–176)	22 (0–88)
Leukaemia (18)	7 (1–146)	0 (0–37)	1 (0–31)	1 (0–105)	0 (0–17)	3 (1–90)	3 (0–82)	3 (0–122)	3 (1–115)	2 (0–24)
Germ Cell (10)	29 (6–559)	0 (0–1)	13 (0–537)	3 (0–525)	8 (0–22)	20 (6–96)	7 (6–88)	29 (0–559)	15 (5–105)	0 (0–20)
Brain / CNS (7)	65 (6–359)	0 (0–90)	40 (0–317)	16 (0–254)	16 (0–33)	33 (1–105)	33 (1–80)	53 (1–359)	42 (1–83)	5 (0–63)
Bone Tumour (7)	86 (45–169)	20 (0–417)	1 (0–80)	1 (0–63)	34 (0–61)	79 (24–168)	30 (24–109)	81 (24–140)	80 (6–169)	21 (2–44)
Melanoma (6)	99 (70–392)	0 (0–0)	31 (0–295)	1 (0–368)	11 (7–15)	84 (24–100)	69 (13–93)	31 (15–295)	69 (39–111)	69 (39–111)
Soft Tissue Sarcoma (5)	41 (20–183)	1 (1–13)	7 (0–38)	0 (0–70)	14 (0–14)	41 (20–113)	27 (18–99)	31 (11–125)	34 (18–145)	13 (0–58)

Note that the median duration for each interval is calculated separately and the components cannot be summated to equal the Total Interval shown in the table

#### Patient interval

Time from first symptom to first presentation was generally very short (Table 5) other than for bone tumour, carcinoma and lymphoma. Panel analysis suggested that symptoms in some patients with these diagnoses could have led to an earlier recognition of cancer.

#### Time in primary and secondary care

Figure 5b illustrates the relative time spent in primary and secondary care (detailed data from Table 5). The primary care interval was greatest in brain/CNS tumours (median 16 days), carcinoma (15 days) and lymphoma (10 days). The time spent in secondary care (i.e. from referral to start of treatment) also varied and was shortest in leukaemia (median 3 days) but longest in melanoma (median 84 days), bone tumour (79 days), carcinoma (53 days), and lymphoma (45 days).

Patients with bone tumour spent very little time in primary care (median 1 day) although they had one of the longest Total Intervals (median 86 days). Pathway analysis suggested that prompt referral to secondary care, frequently to musculoskeletal clinics, accounted for this despite lack of a suspicion of cancer.

Patients with melanoma had the longest Total Interval (median 99 days) but also spent very little time in primary care. This was accounted for by a long Treatment Interval because the start of treatment was defined as the date of wide local excision rather than that of the excision biopsy undertaken after a (short) Referral Interval.

Other than melanoma, patients with carcinoma, lymphoma, brain/CNS tumours and bone tumours had the longest Specialist Care Interval (i.e. from the time of first specialist visit to start of treatment – median 48, 36, 33 and 30 days respectively). These patients were often subject to sequential investigation and/or repeated MDT discussion. Figure 5c highlights the relative times spent awaiting a diagnosis from the time of first healthcare contact (Diagnostic Interval) and before starting treatment (Treatment Interval). Patients with carcinoma, bone tumour and lymphoma all experienced intervals of approximately 3 weeks before starting treatment (median Treatment Intervals 22, 21 and 18 days respectively). Treatment Intervals were shortest in leukaemia and in germ cell tumours (orchidectomy providing a simultaneous diagnostic and treatment event in 9/10).

### General themes

Panel discussion highlighted a number of general themes about patient management including: uncertain accountability for, and ineffective on-going management of, patients within secondary care pathways; timeliness of radiological reporting and delayed response to positive investigations; and non-adherence to National Institute of Clinical Excellence (NICE – now the National Institute for Health and Care Excellence) referral guidance [35]. Concern was also identified about the impact of timely decision-making on patient experience and lack of consistent oversight in the management of pain and other symptoms prior to diagnosis. It was clear that a number of patients continued to rely on primary care for clarification of the management plan despite being in secondary care.

### Final outcome (“Clinical Bottom Line”)

All pathways were assessed in line with NCEPOD definitions (Fig. 1), 94%(98/104) were considered informative of which 40% represented good or best practice; 44% as requiring room for improvement (i.e. that aspects of clinical and/or organisational care could have been better); and 16% as less than satisfactory. This varied between diagnostic groups. No bone tumour pathway and fewer than 30% of lymphoma, brain/CNS and carcinoma pathways were classified as good/best practice. In comparison, 78% of leukaemia pathways and 67% of germ cell tumour pathways were deemed to illustrate good/best practice.

## Discussion

This study confirms the complexity and variability of the diagnostic pathways traversed by TYA with cancer, with differences within and between different diagnostic groups. The proportion of different diagnoses included in the analysis broadly reflects the pattern of disease in the TYA age range although there were fewer than expected brain/CNS tumour patients in the cohort, the reason for which was unclear. This may have represented variability in the incidence of brain/CNS tumours diagnosed and / or referred to the TYA service during the period of the study.

Although the majority of patients presented promptly to primary care with symptoms relating to their cancer, patients with carcinoma, lymphoma and bone tumours experienced longer patient interval suggesting that, in retrospect, diagnosis might have been recognised earlier. Many consulted frequently, some even after referral had been made to secondary care. Overall, cancer was suspected in 34% patients at the time of their first presentation. This was highest for germ cell tumour, melanoma and lymphoma but suspicion was low for those subsequently diagnosed with carcinoma, perhaps as a result of the heterogeneity of the sites involved. It was also low in those with brain/CNS tumours and was not considered at all in any of the patients subsequently diagnosed with bone tumour. This is consistent with evidence that TYA with bone and brain tumours are amongst those most likely to experience longer times to diagnosis [2, 13, 24].

In considering RTD, contrary to other data suggesting low TWW usage in the TYA age group [27], 45% of patients were referred by this route including all of those with melanoma and more than half of all those with carcinoma, lymphoma and soft tissue sarcoma. High use of emergency referral was confirmed (38% overall), including nearly all patients with leukaemia (appropriate for this diagnosis). Excluding leukaemia, 26% of patients with other diagnoses presented via emergency referral but, overall, the review panel felt that 37% of diagnoses identified after emergency presentation could have been made earlier by another route.

The total pathway was seen to vary considerably within and between the diagnostic groups but exceeded 2 months for all except those with leukaemia, which usually presents with rapidly evolving symptoms and signs, germ cell tumour for which suspicion of cancer was high at first presentation, and soft tissue sarcoma - a small group characterised by disease at different sites for which it was difficult to draw specific conclusions.

Despite having high rates of suspicion of cancer at referral, those subsequently diagnosed with melanoma and lymphoma also had long total pathways. The duration of the pathway for melanoma may be seen as an artefact resulting from the use of the date of wide local excision as the date of definitive treatment.

Overall, the data suggest that many patients in diagnostic groups typical of the TYA age group do not pass through the secondary care pathway as smoothly as might be hoped or expected and suggest a need for greater focus on time spent in secondary care before diagnosis and start of treatment. This particularly applied to patients with lymphoma, carcinoma and bone tumours, groups with the longest diagnostic and treatment intervals. Patients with bone tumours also had the longest referral interval, the majority of which was accounted for by the duration of the period between referral to secondary care and review by a specialist after which the diagnosis was considered and specifically investigated.

By way of an example, patients with lymphoma, the most common form of cancer in TYA (and the largest subset in the study) were shown to have a relatively short primary care interval and a high suspicion of cancer at first presentation but then spent a median of 36 days from first specialist consultation, and 18 days from diagnostic biopsy, to start of treatment. Panel review identified examples of unnecessary delay including difficulty in accessing and reporting radiological investigations (for example, reporting an abnormal chest x ray and referring the patient back to the initial clinician to order a CT scan when it was clear that this would be required) – reflecting a more general concern about earlier access to appropriate diagnostics identified in the literature relating to earlier cancer diagnosis [3, 13]. Other concerns included the need for open biopsy after failure of fine needle aspiration cytology to establish diagnosis and transfer between different MDTs (for example referring from head and neck MDT to haematology MDT without progressing required investigations such as a PET CT scan despite confirming the diagnosis of Hodgkin’s lymphoma).

There are weaknesses in this study, as there are in methodologies used by early diagnosis researchers in general [36, 37]. Specifically, we recognise that 11% of patients eligible for approach about participation in this study were excluded on the advice of their local nurse specialist. No information was collected about the reasons and the judgement of the nurses was accepted when determining whether it was reasonable to approach a patient over a study which could raise concerns about time taken to achieve diagnosis. It was therefore not possible to characterise details of this group in relation to those who were approached. Individual diagnostic groups were relatively small and the conclusions reached may not be completely transferable to other settings.

The project was not designed either to relate TTD or RTD to outcome or to capture the young people’s perspectives on their diagnostic experience. TYAs’ interpretation of their RTD may differ from that of the professionals involved and some may benefit from the opportunity to discuss their pre-diagnosis journey either with their GP or another relevant healthcare professional. This could be important in re-establishing relationships to support positive experience in future healthcare encounters.

## Conclusions

In contrast to previous concerns about the need to improve the recognition of alert symptoms and reduce time to referral from primary care, there was little evidence of consistent delay in primary care and patterns of repeated consultation seen after the date of referral and/or diagnosis suggest that support and safety netting was being offered. Achieving timely diagnosis and a prompt start to treatment is dependent both on early referral to, and efficient management in, secondary care. Added to this are the challenges young people report in navigating healthcare systems, and there is a case to be made for improving advocacy, support and pathway management for young people facing a possible diagnosis of cancer.

## Data Availability

Access to original data and analysis can be requested from the Corresponding Author.

## Abbreviations

A & E: Accident & Emergency
ACE: Accelerate, Coordinate, Evaluate
CNS: Clinical Nurse Specialist
GP: General Practitioner
NCEPOD: National Confidential Enquiry into Patient Outcome and Death
NICE: National Institute of Clinical Excellence – now the National Institute for health and Care Excellence
RTD: Routes To Diagnosis
TTD: Time To Diagnosis
TWW: Two Week Wait
TYA: Teenagers and Young Adults

## Footnotes

1 A Caldicott Guardian is a senior person responsible for protecting the confidentiality of people’s health and care information and making sure it is used properly. In the United Kingdom, all NHS organisations must have a person in such a role.

2 An NHS Trust is an organisational unit within the National Health Service in England, generally serving either a geographical area or a specialised function (e.g. a hospital). In any particular location, there may be several trusts involved in the different aspects of healthcare for its residents.
